# Integrating Machine Learning in Clinical Practice for Characterizing the Malignancy of Solitary Pulmonary Nodules in PET/CT Screening

**DOI:** 10.3390/diseases12060115

**Published:** 2024-06-01

**Authors:** Ioannis D. Apostolopoulos, Nikolaos D. Papathanasiou, Dimitris J. Apostolopoulos, Nikolaos Papandrianos, Elpiniki I. Papageorgiou

**Affiliations:** 1 Department of Energy Systems, University of Thessaly, Gaiopolis Campus, 41500 Larisa, Greece; npapandrianos@uth.gr (N.P.); elpinikipapageorgiou@uth.gr (E.I.P.); 2 Department of Nuclear Medicine, University Hospital of Patras, 26504 Rio, Greece; nikopapath@upatras.gr (N.D.P.); dimap@med.upatras.gr (D.J.A.)

**Keywords:** solitary pulmonary nodules, machine learning, positron emission tomography, computerized tomography

## Abstract

The study investigates the efficiency of integrating Machine Learning (ML) in clinical practice for diagnosing solitary pulmonary nodules’ (SPN) malignancy. Patient data had been recorded in the Department of Nuclear Medicine, University Hospital of Patras, in Greece. A dataset comprising 456 SPN characteristics extracted from CT scans, the SUVmax score from the PET examination, and the ultimate outcome (benign/malignant), determined by patient follow-up or biopsy, was used to build the ML classifier. Two medical experts provided their malignancy likelihood scores, taking into account the patient’s clinical condition and without prior knowledge of the true label of the SPN. Incorporating human assessments into ML model training improved diagnostic efficiency by approximately 3%, highlighting the synergistic role of human judgment alongside ML. Under the latter setup, the ML model had an accuracy score of 95.39% (CI 95%: 95.29–95.49%). While ML exhibited swings in probability scores, human readers excelled in discerning ambiguous cases. ML outperformed the best human reader in challenging instances, particularly in SPNs with ambiguous probability grades, showcasing its utility in diagnostic grey zones. The best human reader reached an accuracy of 80% in the grey zone, whilst ML exhibited 89%. The findings underline the collaborative potential of ML and human expertise in enhancing SPN characterization accuracy and confidence, especially in cases where diagnostic certainty is elusive. This study contributes to understanding how integrating ML and human judgement can optimize SPN diagnostic outcomes, ultimately advancing clinical decision-making in PET/CT screenings.

## 1. Introduction

Non-small-cell lung cancer (NSCLC) constitutes a significant portion of cancer-related mortalities worldwide [1]. Among the diagnostic challenges encountered in NSCLC management, the identification and characterization of solitary pulmonary nodules (SPNs) present a critical juncture [2]. The mortality associated with SPNs in NSCLC underscores the urgency for a precise and timely diagnosis to guide effective treatment strategies [3].

Conventional diagnostic modalities, including computed tomography (CT) and positron emission tomography (PET), have become indispensable tools in the initial assessment of SPNs [4]. In most instances, malignancy or benignity is ascertained through just a follow-up CT or PET examinations, without invasive procedures. However, despite their widespread use, these imaging techniques often encounter hurdles in accurately distinguishing between benign and malignant nodules [5,6,7]. The ambiguity inherent in SPN diagnosis manifests as inconclusive test results, yielding high levels of uncertainty and necessitating further invasive procedures for a definitive confirmation.

Amidst these diagnostic challenges, Machine Learning (ML) presents a promising avenue for augmenting clinical decision-making in NSCLC management [8,9,10,11,12]. ML, a branch of artificial intelligence (AI), empowers computer systems to learn from data patterns and iteratively improve performance without explicit programming. By leveraging large datasets encompassing diverse patient profiles and imaging features, ML algorithms hold the potential to discern subtle patterns in SPN characteristics that elude human perception [13,14].

Nevertheless, integrating ML into clinical practice is not without its obstacles. Challenges such as data heterogeneity, algorithm interpretability, and generalizability across diverse patient cohorts pose significant hurdles to the widespread adoption of ML-based diagnostic tools [11,12,15]. Moreover, the reliance on retrospective datasets may inadvertently perpetuate biases and limit the real-world applicability of the developed models.

A prominent limitation in current research is the lack of human expertise within the ML framework [16]. While numerous ML models have been proposed for SPN classification [15,17,18,19,20,21,22], few studies have juxtaposed their performance against that of experienced clinicians. Furthermore, the scarcity of models incorporating expert opinions as a foundational component impedes refining ML algorithms to align with clinical intuition and practice.

In light of these challenges, this paper explores the potential of ML in enhancing the diagnosis of SPNs in NSCLC while addressing the pertinent issues impeding its seamless integration into clinical workflows.

## 2. Materials and Methods

### 2.1. Research Methodology

**Figure 1:**
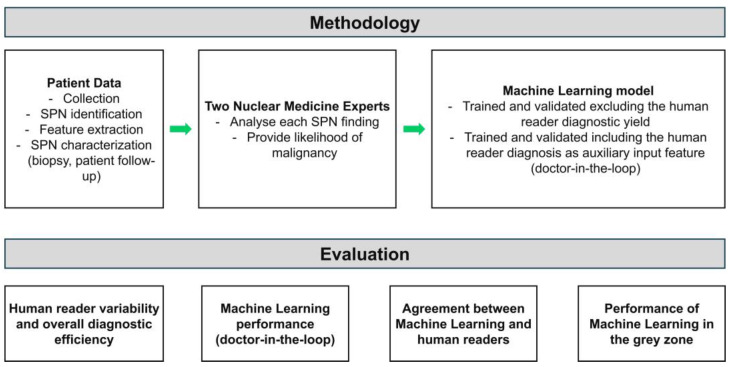
Research methodology.

An overview of the study’s methodology is illustrated in Figure 1. The research employed a dataset encompassing characteristics of SPNs, which is crucial for diagnostic evaluation. These features included dimensions such as diameter, maximum standardized uptake value (SUVmax), spatial location within the lung, and morphological descriptors like margins. Additionally, each SPN entry in the dataset was accompanied by its true label, determined through histopathological analysis, biopsy, or clinical follow-up, categorizing the nodules as benign or malignant.

To emulate the real-world clinical scenario, two independent human readers, blinded to the true labels of the SPNs, provided their subjective assessments regarding the likelihood of malignancy for each nodule. Utilizing a six-point scale ranging from “Highly unlikely” to “Highly likely”, the readers assigned a probability score to each SPN based on their visual interpretation of the imaging features. This yielded a comprehensive perspective of the diagnostic efficiency of human experts in SPN characterization.

ML models were trained and evaluated using the annotated SPN dataset, with and without incorporating human expert opinions as features. Employing ML, the Random Forest (RF) model was designed to predict the likelihood of malignancy for SPNs based on the tabulated features.

First, the diagnostic efficiency of human experts was scrutinized to understand their ability to predict SPN malignancy. Metrics such as accuracy, sensitivity, specificity, and the area under the receiver operating characteristic curve (AUC-ROC) were computed to gauge the reliability of human readers in this task. This assessment provided insight into the extent of human expertise in SPN characterization and served as a benchmark for comparing the performance of ML models.

Subsequently, the RF model was evaluated independently and initially trained solely on SPN characteristic features without incorporating human opinion. This analysis assessed the diagnostic accuracy, sensitivity, specificity, and AUC-ROC of the model, providing a baseline understanding of their performance in the absence of subjective clinical assessments.

The RF model was re-evaluated after integrating human opinion scores as additional features into the training dataset. This step aimed to elucidate the impact of incorporating subjective clinical assessments on model performance. By considering human expertise alongside objective imaging features, the models were expected to refine their predictions, potentially enhancing diagnostic accuracy and confidence.

Furthermore, the alignment between ML probabilities and human scores was scrutinized to assess the convergence of computational and clinical assessments in SPN diagnosis. This analysis aimed to determine the extent to which the ML model mirrored human expert opinions, shedding light on the potential synergies between automated algorithms and human intuition in medical decision-making.

Finally, SPNs falling within diagnostic grey zones, characterized by neither highly likely nor unlikely probabilities, were subjected to thorough analysis. This investigation aimed to evaluate RF’s discriminative capability in clinically ambiguous scenarios, providing insights into their performance under real-world diagnostic challenges encountered in NSCLC management. Through these comprehensive evaluations, the research methodology aimed to elucidate the synergistic potential of ML and human expertise in enhancing diagnostic accuracy and confidence in SPN characterization.

### 2.2. Patient Data

Over a period of five years (2018–2022), an exhaustive review of more than 800 PET/CT scans with confirmed SPNs was conducted to identify potential participants. Data were extracted from the University Hospital of Patras, utilizing PET and CT images obtained from a PET/CT scanner (DISCOVERY iQ3 sl16, GE Healthcare) for each patient case. Physicians meticulously analyzed the images and gathered additional patient information. For each case involving SPNs, supplementary features such as diameter, type, margins, and SUVmax uptake were extracted from the PET scans and documented by human readers. Exclusion criteria were applied to scans lacking SPNs or featuring nodules exceeding 3 cm or less than 0.6 cm in diameter, resulting in 456 qualifying PET/CT scans (Table 1).

Confirmation of SPN malignancy was obtained through biopsy/histopathological confirmation or patient follow-up. Informed consent from patients was waived due to the nature of the survey. Each participant contributed one PET scan and one CT scan. The PET/CT scanner facilitated both scans.

Participants had a mean age of 66 years, with 69% male and 31% female. Benign SPNs comprised 49% of identifications, while malignant SPNs accounted for 51%.

Stringent measures were implemented for data collection, encompassing PET, CT, and clinical information, with a steadfast commitment to anonymity. Sensitive DICOM data were promptly removed post-image collection, and clinical data utilized anonymous identification numbers rather than patient names. This ethical approach aligns with the principles of the Declaration of Helsinki, ensuring participant privacy and upholding the highest standards in medical research. All predicting features were extracted retrospectively by an experienced human reader (N.P. of the authors) and by analyzing each individual’s PET and CT scans.

The dataset encompasses five feature groups (14 features) detailed in Table 2, including numerical or categorical variables covering a broad spectrum of image features that medical staff consider for diagnosis.

### 2.3. Data Preprocessing

In the initial phase of preparing tabular data for classification tasks, an extensive array of preprocessing measures was implemented to enhance the dataset’s suitability for subsequent analyses. Key among these measures is the discretization of potential feature values aimed at mitigating inherent inconsistencies in their representation. This step sought to promote uniformity within the dataset, thus facilitating more effective model training efforts.

Feature selection was strategically approached by extracting new features from those bearing categorical values; for example, the “type” feature, categorized into three parts, was transformed into discrete features to enable a more comprehensive exploration of underlying categorical variables during subsequent analysis.

It is worth noting that missing values were intentionally left unaddressed during preprocessing, as modern ML algorithms possess built-in adaptive mechanisms to handle such occurrences autonomously. This decision reflects confidence in the algorithms’ ability to manage data intricacies without explicit intervention during the preparatory stages.

### 2.4. Expert Probability Scores

In order to assess the diagnostic accuracy of human experts in evaluating the malignancy of SPNs, the study incorporated a method whereby two independent human readers, with specialized training in radiology and no prior knowledge of the definitive diagnostic outcomes, assigned probability scores to each SPN. This evaluation was based solely on the visual interpretation of imaging features observed in PET/CT scans. The readers utilized a standardized six-point Likert-type scale to quantify their assessment of each SPN’s likelihood of being malignant. The scale was defined as follows:

1. Highly unlikely to be malignant;
2. Unlikely to be malignant;
3. Possibly not malignant;
4. Possibly malignant;
5. Likely to be malignant;
6. Highly likely to be malignant.

Each SPN underwent analysis by both readers independently to ensure an unbiased assessment. This dual-reading approach minimized the potential for a single reader’s subjective bias influencing the malignancy likelihood estimates. Moreover, the readers were completely blinded to the true labels (benign or malignant) confirmed through histopathological examination or consistent follow-up, which they did not have access to until all assessments were completed.

The expert probability scores for each scan, along with additional data such as SPN diameter, type, margins, and the maximum standardized uptake value (SUVmax), which were recorded during the initial image analysis phase, were utilized as key variables in the study’s statistical analysis. The endpoint was to correlate the expert assessments with the actual outcomes, hence evaluating the diagnostic performance of human readers.

The expert probability scores, collected from the comprehensive review of 456 qualifying PET/CT scans over a five-year period, were crucial in identifying patterns of agreement or discrepancy between human reader evaluations and actual diagnostic outcomes. This aspect of the study aimed to shine a light on the strengths and limitations of relying solely on human expertise in the diagnostic process of lung cancer screening through SPN characterization.

### 2.5. Machine Learning

#### 2.5.1. Random Forest Algorithm

RF belongs to the ensemble learning family, consisting of an ensemble of decision trees trained on bootstrapped samples of the dataset [23,24]. Each decision tree in the forest is constructed by recursively partitioning the feature space based on predictor variables, aiming to minimize impurity or maximize information gain at each split [25]. However, RF introduces randomness in two key aspects: feature selection at each split and sample selection for training each tree. This randomness mitigates overfitting and enhances the robustness of the model by promoting diversity among constituent trees.

In SPN classification, feature selection significantly identifies informative predictors for distinguishing between benign and malignant SPNs. Random Forest employs a recursive partitioning strategy, where candidate predictor variables are evaluated at each node to maximize information gain or minimize impurity. This iterative process continues until terminal nodes, or leaves, are reached, generating decision rules for nodule classification based on feature thresholds. By integrating a diverse set of features such as nodule diameter, SPN type, and SUVmax uptake (Table 2), RF effectively captures complex relationships within the data and optimally partitions the feature space to enhance predictive performance.

RF employs bootstrap sampling to create diverse training datasets for each decision tree. Bootstrap sampling involves randomly selecting samples from the original dataset with replacement, resulting in multiple subsets of data with potentially overlapping observations. Each decision tree is trained on a bootstrap sample of the data, enabling variability in the training instances and fostering model robustness against noise and outliers.

During tree construction, each decision tree recursively partitions the feature space based on the selected subset of features. The algorithm evaluates different feature thresholds at each split node to optimize a splitting criterion, such as Gini impurity or information gain. The process continues until a stopping criterion is met, such as reaching a maximum tree depth or minimum samples per leaf. Decision trees learn hierarchical decision rules for classifying instances into distinct classes (e.g., benign or malignant SPNs) by iteratively partitioning the feature space.

Once all decision trees are trained, predictions from individual trees are aggregated using a voting scheme to make the final classification decision. In the case of binary classification (benign vs. malignant SPNs), each tree’s prediction is treated as a “vote,” and the class with the most votes across all trees is selected as the final prediction. This ensemble-based approach leverages the collective wisdom of multiple decision trees, resulting in more robust and reliable predictions compared to any single tree.

Hyperparameter tuning is crucial for optimizing RF’s performance and generalization capacity. Grid search, a systematic approach to hyperparameter optimization, exhaustively searches through a predefined hyperparameter space to identify the optimal configuration that maximizes model performance metrics. In the context of Random Forest, hyperparameters such as the number of trees in the forest, maximum tree depth, and minimum samples per leaf significantly influence model complexity and predictive accuracy. Grid search systematically evaluates various hyperparameter combinations through cross-validation techniques, such as k-fold cross-validation, to mitigate overfitting and enhance model robustness.

#### 2.5.2. Probability Calibration

In classification tasks, ML models often provide predicted probabilities indicating the likelihood of different classes for a given instance. However, these predicted probabilities may not be well-calibrated, meaning they do not accurately reflect the true likelihood of class membership. Calibration ensures that predicted probabilities align closely with the actual probabilities, enhancing the interpretability and reliability of the model’s predictions. This is particularly crucial in applications where decision thresholds are sensitive to the relative certainty of predictions, such as medical diagnosis or risk assessment.

**Figure 2:**
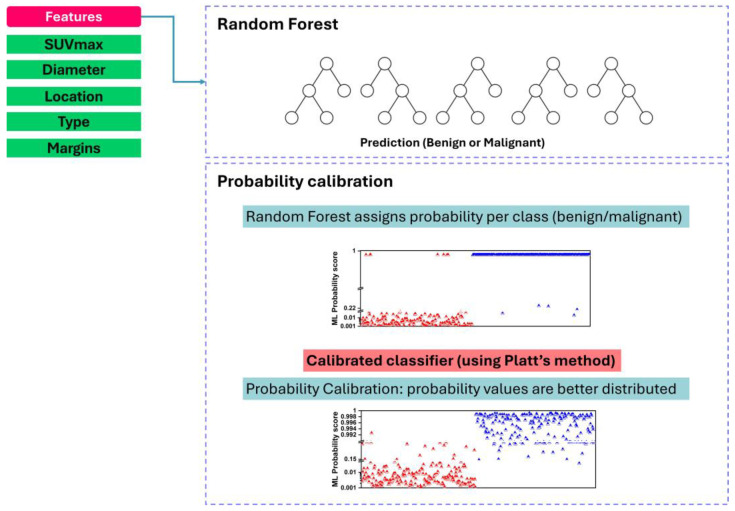
Random Forest and probability calibration process.

Following the classification stage using RF, probability calibration can be performed to refine the predicted probabilities generated by the model (Figure 2). To provide further clarification, our decision to implement sophisticated probability calibration techniques was also driven by an observed issue in the initial outputs of our RF model. Specifically, the model’s probability scores were frequently skewed—either too close to 1 or too close to zero. This skewness represents a significant challenge in the context of our study, where these probability scores needed to be directly comparable with the six-point scale scores provided by human experts. Unlike the more extreme natural distribution of the RF model’s outputs, the expert scores span the entire range from 0 to 1, effectively utilizing the full spectrum of the probability space to classify each SPN.

Platt scaling, a popular calibration method, involves using logistic regression to fit a sigmoid function to the model outputs. This technique adjusts the prediction outputs so that they equate to true probabilities. It is particularly advantageous when the raw outputs of a classifier, typically from models like Support Vector Machines or ensemble methods like RF, are not naturally interpretable as probabilities.

In our study, even though RF provides a natural output that can be interpreted as probabilities, these might still not be well-calibrated. Implementing Platt scaling helps in adjusting these outputs, ideally suited for scenarios where outputs underestimate or overestimate the likelihood of positive classes. Unlike Platt scaling, isotonic regression does not assume any specific functional form between the raw scores and true probabilities. Instead, it employs a piecewise non-parametric approach that is flexible enough to model more complex relationships. Isotonic regression aligns the model outcomes with actual probabilities by fitting a non-decreasing function. We use isotonic regression for cases where the relationship between model outputs and probabilities is suspected to be non-linear. It serves as an effective method when larger datasets are available, reducing the risk of overfitting that this flexibility might otherwise introduce.

In this overview, we focus on implementing probability calibration using the CalibratedClassifierCV class from scikit-learn, which facilitates calibration via sigmoid or isotonic regression.

Probability calibration is often performed within a cross-validation framework to ensure robustness and generalization capacity. In the context of RF classification, probability calibration is conducted separately at each fold of a k-fold cross-validation scheme. This involves splitting the dataset into k-folds, training the RF classifier on k-1 folds, and calibrating predicted probabilities on the held-out fold. By repeating this process for each fold, probability calibration is performed comprehensively across the entire dataset, enhancing the reliability of calibrated probabilities.

In practice, 10-fold cross-validation is commonly employed to evaluate the performance of Machine-Learning models, including Random Forest. In conjunction with probability calibration, each fold of the 10-fold cross-validation involves the following steps:

- Splitting the dataset into ten roughly equal-sized folds;
- Iterating through each fold as the validation set while training the Random Forest classifier on the remaining nine folds;
- Generating predicted probabilities for the validation set using the trained RF classifier;
- Applying probability calibration (e.g., sigmoid calibration) to the predicted probabilities of the validation set, ensuring calibration within each fold;
- Repeating steps 2–4 for each fold to obtain calibrated probabilities for the entire dataset.

By performing probability calibration separately at each fold of the 10-fold cross-validation, the RF classifier’s predicted probabilities are refined to better approximate the true probabilities of class membership. This enhances the model’s reliability and facilitates more informed decision-making in downstream applications.

In the methodology of this study, ML experiments were repeatedly performed, totalling twenty-five iterations for each individual test. This approach was followed to ensure the mitigation of the potentials of random errors and arbitrary possibilities inherent in singular trials. Therefore, this methodologically punctilious approach successfully generated a series of probabilistic outcomes rather than limiting us to deterministic point predictions. Based on such repeated experiment result ranges, we calculated confidence interval (CI) scores, encapsulating the likely range in which our true population parameter lies with a specific level of confidence. A significant feature reflective of our study’s methodological robustness, these CI scores echo our evidentiary weight and sharpness of statistical precision.

## 3. Results

### 3.1. Human Experts’ Diagnostic Yield

Two experienced Nuclear Medicine readers examined each case and provided a malignancy likelihood using a six-point scale (highly unlikely, unlikely, slightly unlikely, slightly likely, likely, and highly likely). The six-point scale was transformed into a binary verdict to assess their reports’ diagnostic efficiency, with the first three points corresponding to benign and the last three to malignant labels.

The two experts scored 90.13% and 87.71% (Table 3). Human Reader 1 correctly identified 203 instances (TP) while incorrectly labelling 14 instances (FP), with 208 true negative cases (TN) and 31 false negatives (FN). This resulted in an accuracy of 0.9013, with a sensitivity of 0.8675 and a specificity of 0.9369. On the other hand, Human Reader 2 also identified 203 true positives but had a higher false positive count of 25. They correctly identified 197 true negatives and missed 31 instances. Consequently, their accuracy was slightly lower at 0.8771, with the same sensitivity of 0.8675 and a specificity of 0.8873. While both readers exhibited comparable sensitivity levels, Human Reader 1 demonstrated slightly superior performance in terms of accuracy and specificity compared to Human Reader 2.

### 3.2. Performance of Machine Learning without Expert’s Verdict

We validated ML using the original predictors from the dataset, i.e., without incorporating the human readers’ opinions when building the model. ML achieves an accuracy of 0.9295 (CI 95%: 0.9287–0.9303), reflecting a high degree of overall correctness in its predictions. Sensitivity and specificity measures are notably robust, with scores of 0.9171 (CI 95%: 0.9156–0.9186) and 0.9426 (CI 95%: 0.9402–0.9449), respectively, underscoring its adeptness in identifying malignant SPNs while minimizing false positives. Precision, as denoted by a score of 0.944 (CI 95%: 0.9418–0.9461), further indicates the model’s ability to accurately classify malignant cases among those predicted as positive, thus enhancing clinical decision-making. Additionally, the model demonstrates a high recall rate of 0.9171 (CI 95%: 0.9156–0.9186), ensuring the capture of true-positive instances among all actual positive cases. The AUC attains a score of 0.979 (CI 95%: 0.9787–0.9794), indicating excellent discrimination between benign and malignant SPNs across varying thresholds.

ML performed slightly better than human readers, reflecting an excellent fit to the training-validation data.

### 3.3. Performance of Machine Learning When Using Human Readers’ Diagnostic Yield as Additional Input

Combining Machine Learning (ML) algorithms with human diagnostic expertise as auxiliary features has proven highly effective in discerning whether SPNs are benign or malignant, as illustrated in Table 4. Moreover, the latter approach was better than building the ML model without the human readers’ opinions (Table 5).

The ML model achieves an impressive accuracy score of 0.9539 (CI 95%: 0.9529–0.9549), indicating its ability to make correct predictions most of the time. It also demonstrates strong sensitivity (0.9688) in identifying malignant SPNs and specificity (0.9383) in recognizing benign ones. We observed an increase in accuracy (+0.0244), followed by analogous improvements in the accompanying metrics when the particular ML strategy was followed (Table 4).

The precision score (0.943) showcases its skill in labelling malignant cases correctly, while the recall rate (0.9688) highlights its ability to capture true-positive instances effectively. Furthermore, the model’s F1 score (0.9557) emphasizes its balanced performance in classifying SPNs. With a high area under the curve (AUC) of 0.992, the model shows exceptional discrimination and agreement between predicted and observed outcomes. Overall, this integration of ML with human insights offers a promising avenue for enhancing the accuracy of SPN classification, potentially improving patient care in clinical settings.

**Figure 3:**
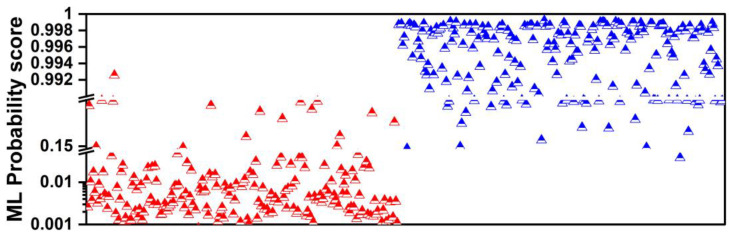
ML probability score distribution between the benign class (red) and the malignant class (blue).

The probability scores of the ML model are illustrated in Figure 3. Each triangle represents a unique patient case. Red triangles denote true benign SPNs and blue triangles denote true malignant SPNs. For each SPN, the ML probability of the malignancy score is obtained and plotted. In this way, we observe how the assigned probability scores are distributed between the two classes. The reader can notice two distinct probability areas. Most benign-predicted instances show probability scores below 0.15 (15%), whereas the majority of malignant-predicted instances fall well above a 0.992 (99.2%) probability. The latter phenomenon can be attributed to the inherent tendency of the model to overestimate or underestimate the probability scores, even after probability calibration.

### 3.4. Concordance between Machine Learning and Human Readers

**Figure 4:**
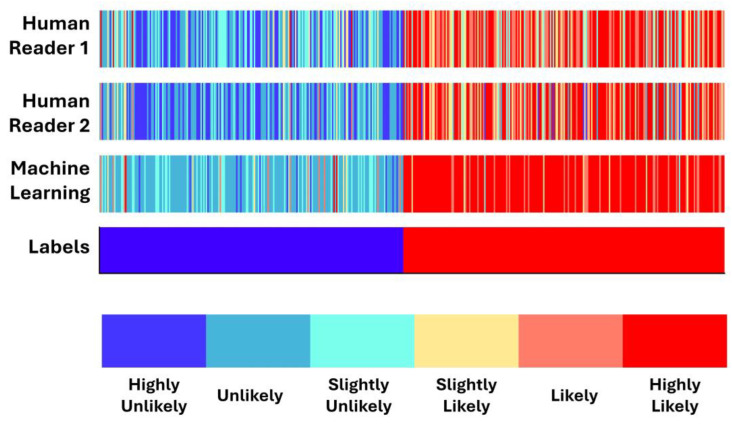
Color bars visualizing each SPN likelihood of being malignant as assigned by the two human readers and by the ML algorithm. SPN cases are spread along the *x*-axis and sorted by label.

**Figure 5:**
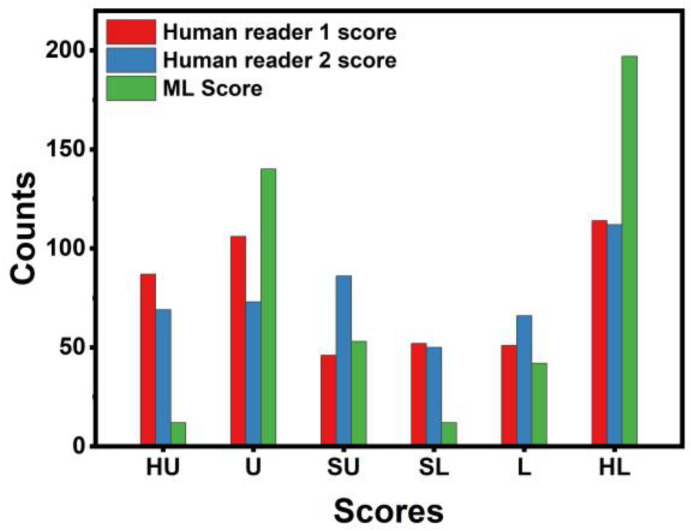
Histogram of probability groups between the two human reader and the ML model. HU, U, SU, SL, L, and HL stand for Highly Unlikely, Unlikely, Slightly Unlikely, Slightly Likely, Likely, and Highly Likely, respectively.

Figure 4 presents color bars visualizing each SPN’s likelihood of being malignant, as assigned by the two human readers and the ML algorithm. SPN cases are spread along the *x*-axis and sorted by label. Approximately half of the bar contains benign SPNs (blue) and half malignant (red). ML assigns more solid scores to malignant SPNs, and benign SPNs, asobserved. Human readers spread their scores among the potential groups (Figure 5). More specifically, we observe that the Highly Unlikely (HU) category is used chiefly by human readers and not by the ML model.

On the contrary, the Unlikely (U) category is populated mainly by the ML model, whilst human readers assigned the majority of benign SPNs. ML and Human Reader 1 assigned an approximately equal number of SPNs in the Slightly Unlikely (SU) class category. Moving to the malignant-oriented categories, the ML model assigned a few SPNs in the category of Slightly Likely (SL). Both human readers and the model assigned an approximately equal number of SPNs in the Likely (L) class. Notably, ML assigned a higher proportion of SPNs in the Highly Likely (HL) class than the human readers.

### 3.5. Performance of Machine Learning in Ambiguous Cases

We evaluated the performance of the ML model in cases where the best human reader (Human Reader 1) was assigned a score between SU and SL. The latter zone is considered the grey zone for the human reader, and ML could be beneficial in SPN cases that fall into this zone.

Table 6 presents the performance of the first human reader and the ML model in the grey zone. Human Reader 1 detected 44 true positives, 8 false positives, 35 true negatives, and 11 false negatives. Their accuracy was 80.61%, sensitivity 80%, and specificity 81.39%.

The ML model detected 53 true positives, 8 false positives, 35 true negatives, and 2 false negatives. Its accuracy was 89.79%, sensitivity 96.36%, and specificity 81.39%. The ML model outperformed Human Reader 1 in accuracy and sensitivity, achieving 89.79% accuracy and 96.36% sensitivity, compared to 80.61% accuracy and 80% sensitivity for the human reader. However, both exhibited the same specificity of 81.39%.

**Figure 6:**
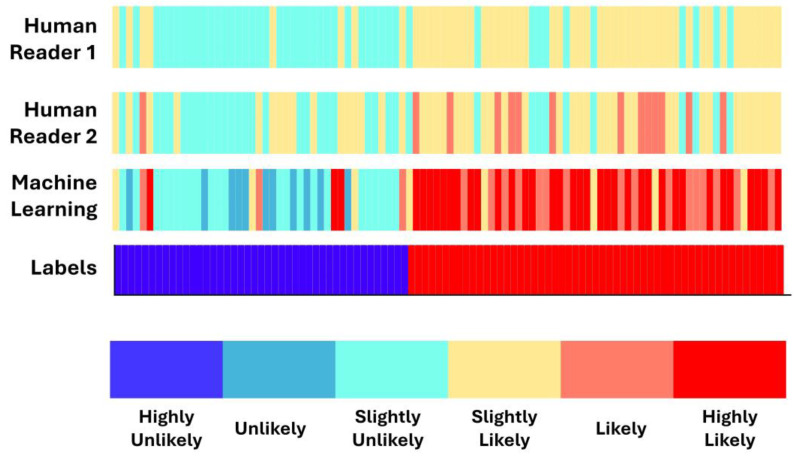
Color bars visualizing each SPN likelihood of being malignant as assigned by the two human readers and by the ML algorithm. SPN cases that are considered ambiguous by the first human reader are spread along the *x*-axis and sorted by label.

Figure 6 presents color bars visualizing each SPN’s likelihood of being malignant as assigned by the two human readers and the ML algorithm. SPN cases considered ambiguous by the first human reader are spread along the *x*-axis and sorted by label.

**Figure 7:**
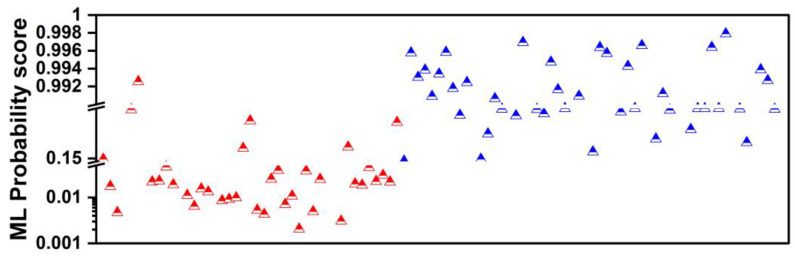
ML probability score distribution between the benign class (red) and the malignant class (blue) in cases considered ambiguous by the first human reader.

We observe a variability between the two human readers in malignant cases. More specifically, SPN instances considered ambiguous by the first reader are graded with different scores from the second. On the other hand, ML estimates the malignant instances with higher probabilities (falling in the categories of L and HL). ML assigns probability scores for benign cases that fall within the U and SU categories (Figure 7). The latter underlines an agreement with the human readers.

### 3.6. Comparison with the Literature

In our comparative analysis of the literature associated with the diagnostic accuracy of various methodologies in the classification of solitary pulmonary nodules (SPNs), our study has demonstrated results that are not only comparable but also highly consistent across different dimensions of analysis. The findings from our study are detailed in Table 7 below, alongside a comprehensive range of results derived from other prominent studies within the field.

When juxtaposed with results reported in the literature where studies predominantly utilized either direct imaging modalities such as CT or PET—a few incorporating clinical data—our approach integrating features from both CT and PET images presents several advantages.

Studies [8,11,12,26], primarily using CT images, have shown a high accuracy and sensitivity but varied in specificity. Our method maintains high metrics across all three parameters, underscoring the benefit of utilizing a combination of imaging features, which potentially capture more comprehensive pathological insights.

Comparing with studies like [19,21], which also used PET/CT images but reported lower AUCs (0.876 and 0.81, respectively), our approach highlights the robustness of combining extracted features from these imaging types. Additionally, our results appear superior even when compared to hybrid studies like [20], which integrated PET, CT, and clinical data but showed a lower specificity. Relative to studies employing clinical data alone ([16,18]), which demonstrated the versatility of non-imaging data but yielded a lower accuracy and AUC, our model underscores the importance of image-derived features in enhancing diagnostic accuracy. 

Even when juxtaposed with complex models that incorporate additional parameters like radiomics ([21] with an AUC of 82%), our method provides a competitive edge with a substantially higher accuracy and discriminative power, suggested by our higher AUC.

## 4. Discussion

The present study presented an ML methodology for identifying the malignancy of SPNs in patients undergoing PET/CT screening. We used a dataset containing various characteristics of SPNs crucial for diagnostic assessment, such as size, SUVmax uptake, location, and morphological descriptors. Using a six-point scale, two human readers independently assessed the likelihood of malignancy for each SPN. An ML model was trained and evaluated using this dataset, both with and without incorporating human assessments as features. The diagnostic efficiency of human experts was evaluated first, followed by the assessment of the ML model (RF) trained solely on SPN features. Then, the RF model was re-evaluated after integrating human assessments. The alignment between ML predictions and human scores was examined to assess convergence. Finally, SPNs in diagnostic grey zones were analyzed to evaluate the models’ performance in ambiguous scenarios. The study aimed to understand how ML and human expertise could synergize to enhance diagnostic accuracy and confidence in SPN characterization.

We can summarize the findings of the experiments as follows:

(a) Human readers exhibited variability when providing their assessments. The latter was reflected in the different performance scores (first reader: ~90% accuracy; second reader: ~88% accuracy).
(b) Integrating both readers’ diagnostic scores in the training features of the ML model resulted in improving the diagnostic efficiency of the ML model by ~3%. The latter underlines the importance of the synergistic contribution of ML and human judgement in discriminating between benign and malignant SPNs.
(c) ML assigns probability scores that swing too high or too low, whereas human readers excel in carefully identifying ambiguous cases. The latter is better observed in Figure 4 and Figure 5.
(d) ML performed better than the best human reader in challenging instances (i.e., SPNs with probability grades of Slightly Unlikely and Slightly Likely). ML produced 2 FNs, whilst the best human reader had 11 FNs. The latter is reflected in the overall performance of ML in the grey zones (~90% accuracy, 80% sensitivity, and 81.39% specificity). Hence, ML could be particularly useful in such cases where diagnostic yields are challenged.

The superior performance of the ML algorithm compared to human readers in classifying SPNs as benign or malignant, as evidenced by the comprehensive suite of performance metrics, underscores the potential of computational methodologies in medical diagnostics. 

However, it is essential to interpret this discrepancy judiciously within the context of the specific dataset utilized for model training and evaluation. ML algorithms excel in discerning intricate patterns and relationships within large and complex datasets, leveraging mathematical algorithms to refine their predictive capabilities iteratively. In contrast, while invaluable, human diagnostic expertise may be influenced by cognitive biases, variability in interpretation, and limitations in scalability and consistency. Moreover, the performance of ML models on unseen data, a crucial benchmark for assessing generalizability, remains a pertinent consideration. The study lacks an independent validation set to verify the applicability of the methodology to other Nuclear Medicine laboratories. 

In real-world clinical scenarios, the heterogeneity and unpredictability of patient cases may pose challenges for ML algorithms, particularly in instances where nuanced clinical judgment and domain-specific knowledge are indispensable. Therefore, while ML demonstrates remarkable efficacy within the confines of the training dataset, its performance in extrapolating to novel, real-world contexts may be tempered by inherent limitations, including the lack of clinical expertise and contextual understanding. Thus, a valid perspective that acknowledges both the strengths and limitations of ML in medical diagnostics is imperative for its responsible integration into clinical practice, where human judgment and computational prowess synergistically contribute to optimized patient care.

A notable limitation in our study is the absence of a detailed analysis of morphological characteristics, location, and grayscale data of SPNs. Our reliance on pre-extracted, quantifiable features from clinical reports, rather than structured imaging data, constrains the depth of our findings. Specifically, the procedure deployed is susceptible to individual bias, veering slightly away from the principle of replicable scientific objectivity. Alternative, less subjective systems of feature extraction exist. For instance, open-source software, such as 3D Slicer equipped with the Pyradiomics package, can automatically and more uniformly extract salient features from CT images of SPNs even including morphological and radiomics characteristics accessed from the contours, directions, and gray values.

It is important for future research to incorporate these detailed imaging parameters to provide a more comprehensive understanding of SPN characteristics that might enhance diagnostic accuracy.

## 5. Conclusions

This study demonstrates that integrating ML methods into clinical practice for diagnosing SPN malignancy can significantly enhance diagnostic accuracy. The findings indicate that the ML models were more effective when a doctor’s assessment was included as an additional feature, suggesting the potential value of AI–human collaboration in SPN diagnosis and management. Human readers exhibited variability in their assessments, showcasing discernment, particularly in ambiguous cases. Integrating human judgement alongside ML significantly enhanced diagnostic efficiency, demonstrating a synergistic relationship between the two approaches. While ML outperformed human readers in challenging instances, particularly in identifying ambiguous cases, its tendency to swing probability scores too high or too low highlights the complementary strengths of human judgment. Ultimately, the findings suggest that a collaborative approach, leveraging the strengths of both human and machine intelligence, is paramount in accurately discriminating benign and malignant SPNs, especially in cases where diagnostic certainty is elusive.

## Figures and Tables

**Table 1:** Information on the study’s dataset.

Clinical Characteristics	Frequency
No. of participants	456
Age (mean ± sd)	66 ± 8
Sex (male/female)	69% male/31% female
Total benign SPNs	222 (48.6%)
Total malignant SPNs	234 (51.4%)

**Table 2:** Details of the dataset’s features.

Feature Name	Type of Feature	Type of Values	Potential Values
SUVmax	FDG Uptake	Numeric	
Diameter	SPN Feature	Numeric	0.6 to 3 cm
Location	SPN Feature	Categorical	Left Lower Lobe (LLL) Lingula Middle Right Upper Lobe (RUL) Right Lower Lobe (RLR)
Type	SPN Feature	Categorical	Solid Semi-solid Ground-class
Margins	SPN Feature	Categorical	Well-defined Lobulated Spiculated Ill-defined

**Table 3:** Human readers’ diagnostic yields. TP, FP, TN, and FN stand for True Positives, False Positives, True Negatives, and False Negatives, respectively. ACC, SEN, and SPE denote Accuracy, Sensitivity, and Specificity, respectively.

	TP	FP	TN	FN	ACC	SEN	SPE
Human Reader 1	203	14	208	31	0.9013	0.8675	0.9369
Human Reader 2	203	25	197	31	0.8771	0.8675	0.8873

**Table 4:** Machine Learning performance. ACC, SEN, and SPE denote Accuracy, Sensitivity, and Specificity, respectively.

	ACC	SEN	SPE
ML without human diagnosis as input feature	0.9295 (CI 95%: 0.9287–0.9303)	0.9171 (CI 95%: 0.9156–0.9186)	0.9426 (CI 95%: 0.9402–0.9449)
ML with human diagnosis as input feature	0.9539 (CI 95%: 0.9529–0.9549)	0.9688 (CI 95%: 0.967–0.9707)	0.9383 (CI 95%: 0.9373–0.9393)

**Table 5:** Performance metrics of Machine Learning when using the human readers’ diagnostic yields as auxiliary features.

Metric	Score
Accuracy	0.9539 (CI 95%: 0.9529–0.9549)
Sensitivity	0.9688 (CI 95%: 0.967–0.9707)
Specificity	0.9383 (CI 95%: 0.9373–0.9393)
F1	0.9557 (CI 95%: 0.9547–0.9567)
AUC	0.992 (CI 95%: 0.9919–0.9921)
Kappa	0.9078 (CI 95%: 0.9058–0.9098)
TP	227
TN	208
FP	14
FN	7
FPR	0.0617 (CI 95%: 0.0607–0.0627)
FNR	0.0312 (CI 95%: 0.0293–0.033)
PPV	0.943 (CI 95%: 0.9422–0.9439)
NPV	0.9152 (CI 95%: 0.9139–0.9165)

**Table 6:** Human reader and ML diagnostic yields in ambiguous SPN cases. TP, FP, TN, and FN stand for True Positives, False Positives, True Negatives, and False Negatives, respectively. ACC, SEN, and SPE denote Accuracy, Sensitivity, and Specificity, respectively.

	TP	FP	TN	FN	ACC	SEN	SPE
Human Reader 1	44	8	35	11	0.8061	0.8	0.8139
ML	53	8	35	2	0.8979	0.9636	0.8139

**Table 7:** Comparison with the literature.

Study	Data Type	Test Data Size	Results
[12]	CT image	897	ACC: 90.85% SEN: 94.76% SPE: 82.05%
[26]	CT image	1113	ACC: 92.07% SEN: 89.35% SPE: 94.80%
[11]	CT image	112	ACC: 94% SEN: 92% SPE: 94.50%
[10]	CT image	1297	AUC: 0.936
[22]	CT image	208	AUC: 0.85
[15]	CT image	252	ACC: 90.6% SEN: 83.7% SPE: 93.9%
[8]	CT image	2119	ACC: 85.23% SEN: 92.79% SPE: 72.89% AUC: 0.9275
[21]	PET/CT image	48	AUC: 0.81 SEN: 88% SPE: 86%
[19]	PET + CT images	1168	ACC: 79% AUC: 0.876
[20]	PET + CT + Clinical	105	ACC: 85% SEN: 86% SPE: 33%
[27]	PET image	86	ACC: 86% SEN: 64% SPE: 91%
[28]	CT + PET images	270	SEN: 72% SPE: 82%
[18]	CT image	1175	ACC: 74.5% AUC: 0.795
[16]	Clinical data	452	ACC: 86.54%
[17]	CT image + Clinical data	227	AUC: 88.2%
[18]	Clinical data	200	ACC: 75.6% F1: 72.2% AUC: 82%
[15]	CT image	552	ACC: 84.1% AUC: 90.3%
[19]	PET/CT image	55	SEN: 85.2% SPE: 82.1%
[21]	PET/CT image + Radiomics	106	AUC: 82%
This study	CT and PET image features	456	ACC: 0.9539 (CI 95%: 0.9529–0.9549) SEN: 0.9688 (CI 95%: 0.967–0.9707) SPE: 0.9383 (CI 95%: 0.9373–0.9393) AUC: 0.992 (CI 95%: 0.9919–0.9921)

## Data Availability

The datasets analyzed during the current study are available from the nuclear medicine physician upon reasonable request.
